# The Psychology of Kink: A Cross‐Sectional Survey Investigating the Association Between Adult Attachment Style and BDSM-Related Identity Choice in China

**DOI:** 10.1007/s10508-024-02829-1

**Published:** 2024-03-07

**Authors:** Shengyu Li

**Affiliations:** School of Stomatology, Southern Medical University, 510515 Guangzhou, China

**Keywords:** BDSM, Attachment style, BDSM identity, Dom-type, Sub-type, Switch

## Abstract

BDSM is a type of sexual preference that includes bondage and discipline, dominance and submission, and sadism and masochism. Research has identified three specific power exchange roles in the practice of BDSM: dominance, submission, and switch. It has also been suggested that attachment style potentially influences BDSM interests. This study investigated the potential roles of attachment style in driving BDSM identity. A questionnaire was completed by a cross-sectional Chinese sample (*n* = 3310, age range 18–30 years), including 1856 BDSM practitioners (436 men, 1420 women). To assess attachment style, the questionnaire included a Chinese translation of the Adult Attachment Scale as well as items surveying BDSM interests. Compared to non-BDSM practitioners, attachment styles were not significantly different from BDSM practitioners. However, practitioners with different BDSM identities showed a significant difference in their attachment styles. Secure and avoidant attachment styles were associated with dominance, whereas submissiveness recorded high average scores of separation anxiety in both males and females. BDSM identities based on gender revealed that 60.5% of female practitioners assumed the role of submissiveness and this group recorded the highest average scores of separation anxiety among all groups. These results show that BDSM identity is related to attachment style. However, the results did not support the hypothesis that attachment styles potentially drive BDSM identities. Further research is needed to explore other psychological processes that drive BDSM identities in order to provide guidance for BDSM practitioners in choosing suitable identities, thereby helping practitioners to choose suitable identity partners and avoid negative experiences during BDSM participation.

## Introduction

BDSM is an abbreviation for the combination of bondage and discipline, domination and submission, and sadism and masochism. The essence of BDSM is based on the SSC principle (safe, sane, and consensual), a pattern of getting along in which the two parties exchange their power in a safe, rational, and informed manner, i.e., practitioners engage in BDSM activities willingly, whether on a temporary or permanent basis, and agree to cede some of their power to the other party (Jie & Jia, 2017; Stein, 1984). The pursuit of BDSM as a sexual orientation is not indicative of a lack but of a path to self-discovery (Carlström, 2019). In certain dynamics, BDSM practitioners may evolve or change identities during play or over time and carry out activities matching new identities based on the consent of all practitioners (Schori et al., 2022). Practitioners can choose many identities, and the most famous identity categories are based on the following five types: domination, submission, sadism, masochism, and switch. The identities of domination and submission are more inclined to psychological activities, whereas sadism and masochism are more inclined to physical (Richters et al., 2008). In practice, however, the vast majority of practitioners will choose multiple identities, and there are numerous other roles, such as top, bottom, caregiver, little, and more. According to the common characteristics of the identities selected by practitioners, three types are used in this study: Dom-type, Sub-type, and Switch. Dom-type refers to a dominant identity in BDSM activities, including mental and physical dominance, i.e., domination and/or sadism (such as top and caregiver). Sub-type refers to a passive identity in activities and involves a transfer of power, i.e., submission and/or masochism (such as bottom and little). Switch involves a switching of identity between Dom-type and Sub-type under certain conditions, such as the needs of practitioners or the characteristics of peers (Schuerwegen et al., 2021; Wignall & McCormack, 2017). Identity selection is crucial in BDSM, but in practice not all practitioners can choose their own identity. Therefore, studying the factors that affect identity selection can help practitioners who are troubled by identity selection to make decisions.

BDSM is gradually becoming less stigmatized in contemporary society. No research has shown that BDSM practitioners are driven by psychological disorders; hence, it cannot be said that a psychological disorder drives either Dom-type or Sub-type. Although studies have pointed out that BDSM practitioners potentially of Sub-type are more likely to have mental health issues, there is no evidence of a clear link between BDSM identity selection and a mental disorder (Brown et al., 2020). In addition, the results of Richters et al.’s (2008) interviews showed that BDSM practitioners were more attracted by sexual interests or subcultures, and the vast majority had no past history of abuse, and no tendency to suffer adverse sex-related issues. Moreover, Wismeijer and Assen (2013) noted that BDSM practitioners may have better psychological outcomes than non-BDSM practitioners.

In the Chinese Classification and Diagnostic Criteria for Mental Disorders, 3rd edition (CCMD-3), sexual abuse is defined as using abuse or the acceptance of sexual abuse as the primary means of sexual arousal, but sexual abuse is not included in the category of disorders under the premise of informed consent. The paraphilia disorder associated with sexual abuse, as defined in the fifth edition of the *Diagnostic and Statistical Manual of Mental Disorders*, is based on an involuntary premise (Palmer et al., 2018). Therefore, BDSM activity following SSC principles is not a manifestation of mental disorders under this diagnostic criterion.

Although studies have begun to focus on the many factors that drive BDSM interests, no decisive factors have been found, and there is limited evidence of the extent to which BDSM interests are related to various possible factors. Recent studies have pointed out that gender, sexual orientation, attachment style, parenting style, cultural context, and trauma are all related to the tendency to participate in BDSM (De Neef et al., 2019; Ten Brink et al., 2021).

Attachment is defined as the emotional bond created when an individual forms a lasting relationship with others. This emotional bond will affect identity formation and interpersonal beliefs and behaviors. The Adult Attachment Scale (AAS; Collins & Read, 1990) assesses adult attachment styles by an evaluation of three latitudes: closeness, dependence, and anxiety. Closeness refers to the willingness of an individual to connect with others. Dependence refers to the extent to which an individual is willing to rely on others, and anxiety refers to the degree to which an individual is worried about separation from the partner (Mortazavizadeh et al., 2022). By combining the three latitudes, and comparing the average scores of closeness, dependence, and anxiety, attachment styles can be divided into four types: secure, avoidant, anxious, and insecure. Anxious-type individuals tend to show a desire for intimacy, worry about separation, and show low levels of self-identity (Pietromonaco & Barrett, 1997). Avoidant types show a strong preference for self-reliance and the rejection of intimacy. Secure types show high levels of self-identity and a high level of trust in, and affirmation of, the other. Insecure types show low levels of self-identity and difficulties in trusting others (Brennan et al., 1998). Attachment styles can represent how different individuals feel about themselves and their partners in interpersonal relationships, and studies have shown that attachment styles can predict an individual’s quality of life (Darban et al., 2020), interpersonal behavior (Hoenicka et al., 2022), sexual contact, and other behaviors (Tucker et al., 2022). Anxious types self-reported higher negative feelings, stress, and perceived rejection experiences than secure types, while avoidant types showed lower expectations of being alone with others than secure types (Sheinbaum et al., 2015). These results show that different attachment styles may predict different behaviors. In a study of the association of attachment styles with BDSM interests, BDSM practitioners showed more secure and more anxious attachment styles than the non-BDSM practitioners (Ten Brink et al., 2021). Moreover, Coleman et al. (2023) found that anxious-type individuals may be willing to have compulsive sex with others, which is similar to the Sub-type tendency in BDSM. Szielasko, Symons, and Plass found that individuals with avoidant attachment styles may have more sexual partners in their lifetime, while individuals with insecure attachment styles are more likely to perform overwhelming and guiding behaviors (Szielasko et al., 2013), which are common in BDSM Dom-type activities. Therefore, we can assume that attachment style is an important factor that may influence or even predict BDSM identities.

BDSM identity determines the practitioner’s choice of partner, and they choose a partner that matches their identity (Alison et al., 2001). According to Jozifkova (2013), an unsuitable partner identity can lead to a destructive relationship or behavior. If someone who desires power is forced to tolerate abusive behavior, they will eventually lose satisfaction in the relationship. When one partner switches from dominant to submissive, a negative relationship experience can occur if the other partner cannot switch roles. That explains why a switch could be a Dom-type on some occasions and a Sub-type on others, but not necessarily with the same partner. It seems that while switches always play one role within their primary relationship, they may find an external relationship to feed their other needs. Therefore, before BDSM practitioners define their identities, they should define their own self-orientation and expectations of their partners. Attachment styles can help practitioners to better realize their expectations of being close to their partners, their dependence on their partners, and their worries about being abandoned.

The present study sets out to investigate the correlation between attachment styles and the choice of a BDSM identity, the characteristics of attachment styles of different identities, and the influence of different attachment styles on identity choice. It is expected that the results will help practitioners to choose identities more reasonably, and to define their expected partner identities in order to develop benign relationships.

## Method

### Participants

Data were gathered from October 2022 to November 2022 by means of an online questionnaire created on the Wenjuanwang platform (www.wenjuan.com). The questionnaire was distributed among non-BDSM practitioners and BDSM practitioners via a BDSM-related WeChat Official Account (a general social media site). BDSM practitioners could only participate in the study if they gave a positive response to the question, “Are you someone who engages in BDSM-related activities?” Respondents who said “no” to this question were classified as non-BDSM practitioners and treated as controls. In total, 1856 BDSM practitioners and 1454 non-BDSM practitioners participated in the survey.

### Procedure and Measures

Sexual orientation, parenting styles, and sexual abuse were measured by self-identification in response to the following questions: “What is your sexual orientation?”, “What is your parenting style?”, and “Have you ever suffered sexual abuse?”

BDSM practitioners were subdivided into 3 BDSM identities: “Dom-type” (if they prefer discipline and/or dominance and/or act as a sadist and/or act as a caregiver in BDSM-related activities; *n* = 314); “Sub-type” (if they prefer to be submissive and/or be subjected to bondage and/or act as a masochist and/or as a “little” role in BDSM-related activities; *n* = 981); and “Switch” (if can change their identity as they wish; *n* = 561).

To evaluate attachment styles, a Chinese version of the AAS was used (Wei-li et al., 2004). Subjects were asked to score 18 items related to attachment on a 5-point scale with 1 indicating “strongly disagree” and 5 signifying “strongly agree” (see Appendix), with 7 items reverse scored. The mean score was determined by calculating the average score for each of the three latitudes (Closeness, Dependence, and Anxiety). Attachment styles were grouped into four categories, as follows. (1) Secure attachment style with a mean score ≥ 3 on the total Closeness scale and total Dependence Scale and a mean < 3 on the total Anxiety scale. (2) Anxious attachment style with a mean score ≥ 3 on the total Anxiety scale. (3) Avoidant attachment style with a mean score < 3 on the total Closeness scale and total Dependence Scale and a mean score < 3 on the total Anxiety scale. (4) Insecure attachment style with a mean score < 3 on the total Closeness scale and total Dependence scale and a mean score ≥ 3 on the total Anxiety scale.

### Data Analysis

Statistical analysis was performed using SPSS 24.0. Chi-square analyses were used for nonparametric demographic variables. Initial analyses compared the three BDSM subgroups (Dom-type, Sub-type, and Switch) to controls. Finally, univariate analysis of variance was applied to examine correlations between BDSM identities and attachment styles.

## Results

### Demographics

In total, 3538 individuals completed the survey. Of the total, 3310 (93.6%) respondents ranged in age from 18 to 30 years. To control for age difference, later analyses were based on individuals in the 18–30 range. Of the BDSM practitioners, 314 (16.9%) individuals were identified as Dom-type; 981 (52.9%) were identified as Sub-type; and 561 (30.2%) as Switch.

Demographic variables are shown in Table 1. Analysis showed that the majority of male BDSM practitioners were Dom-type, while the majority of Sub-type practitioners were female. Heterosexuals were fewer in each BDSM identity group compared to the control group. The proportion of practitioners in each BDSM identity group with experience of sexual abuse was slightly higher compared to the control group.

**Table 1:** Demographic parameters of non-BDSM practitioners and BDSM practitioners acting out different identities

Demographic variables	Dom-type (*n* = 314)	Sub-type (*n* = 981)	Switch (*n* = 561)	Controls (*n* = 1454)	Test (*p*)
Gender					252.7 (< 0.001)
Male	174 (23.1%)	122 (16.2%)	140 (18.6%)	316 (42.0%)	
Female	140 (5.5%)	859 (33.6%)	421 (16.5%)	1138 (44.5%)	
Sexual orientation					120.6 (< 0.001)
Heterosexual	217 (10.7%)	592 (29.3%)	250 (12.4%)	961 (47.6%)	
Homosexual	16 (8.9%)	52 (29.1%)	50 (27.9%)	61 (34.1%)	
Bisexual	74 (7.1%)	325 (31.2%)	256 (24.6%)	386 (37.1%)	
Asexual	7 (10.0%)	12 (17.1%)	5 (7.1%)	46 (65.7%)	
Parenting styles					10.1 (0.346)
Authoritative	120 (9.6%)	341 (27.2%)	223 (17.8%)	568 (45.4%)	
Authoritarian	86 (9.6%)	289 (32.1%)	159 (17.7%)	366 (40.7%)	
Permissive	56 (9.1%)	184 (30.0%)	96 (15.6%)	278 (45.3%)	
Neglecting	52 (9.6%)	167 (30.7%)	83 (15.3%)	242 (44.5%)	
Sexual abuse					51.2 (< 0.001)
Yes	45 (5.5%)	299 (36.9%)	161 (19.9%)	306 (37.7%)	
No	269 (10.8%)	682 (27.3%)	400 (16.0%)	1148 (45.9%)	

### Attachment Style

**Fig. 1:**
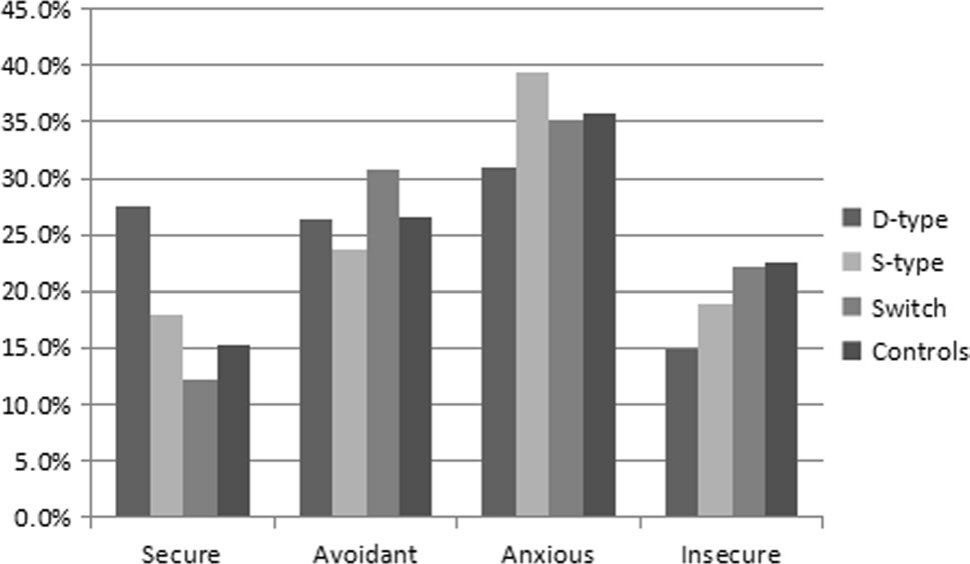
Attachment styles for male BDSM practitioners and non-BDSM practitioners

**Fig. 2:**
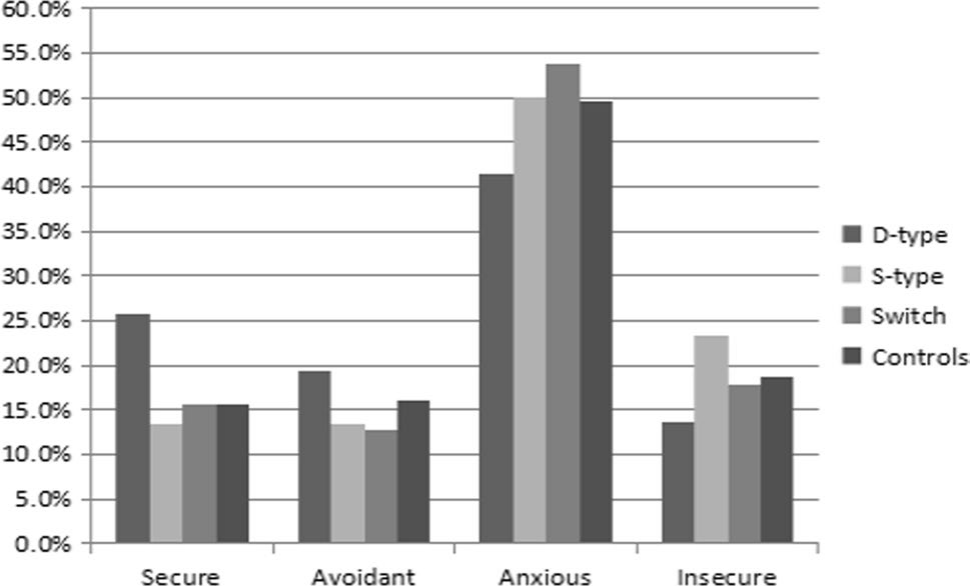
Attachment styles for female BDSM practitioners and non-BDSM practitioners

Attachment styles in the different identity groups and control group were compared. Figures 1 and 2 show the percentages of the different attachment styles in the identity groups and control group. Compared to other groups, the percentages of secure and avoidant attachment styles were higher in the Dom-type group, whereas the percentages of anxious and insecure attachment styles were higher in the Sub-type group. All three groups were significantly different in attachment style according to chi-square tests (male: χ^2^ = 19.3, *p* = 0.023; female: χ^2^ = 30.4, *p* < 0.001).

**Fig. 3:**
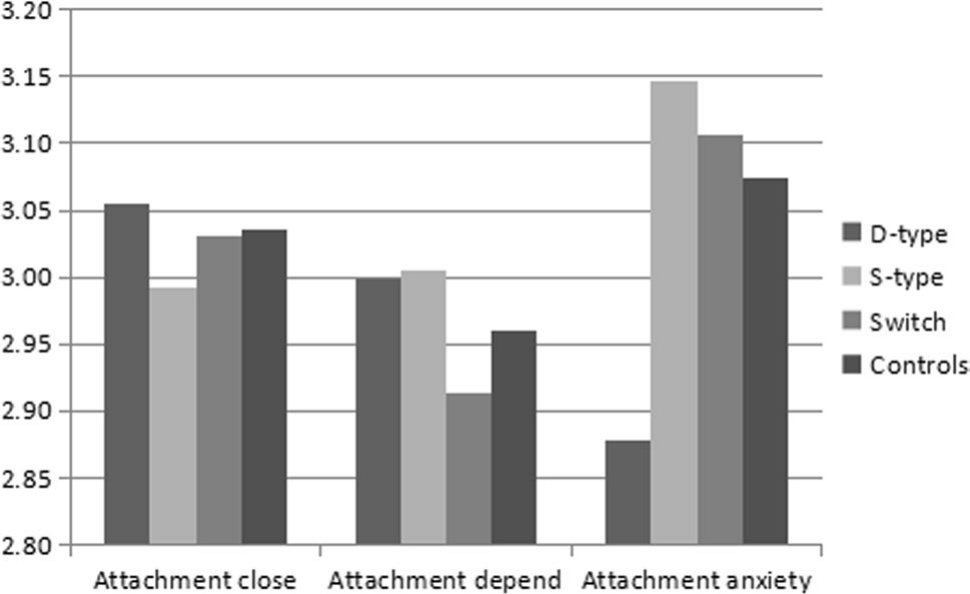
Mean levels of attachment style reported by male BDSM practitioners and non-BDSM practitioners

**Fig. 4:**
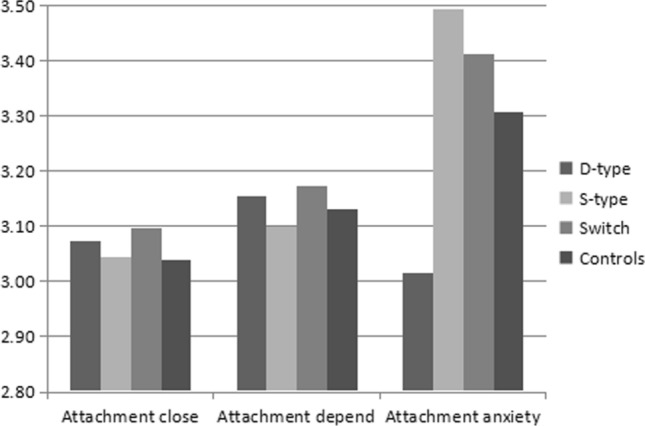
Mean levels of attachment style reported by female BDSM practitioners and non-BDSM practitioners

A multivariate general linear model analysis revealed a significantly higher mean level of anxious attachment styles in the Sub-type group compared to other groups both in male and female respondents, as shown in Figs. 3 and 4 (male: *F* = 2.616, *p* = 0.05, *df* = 3; female: *F* = 15.83, *p* < 0.001, *df* = 3). In contrast, no significant differences emerged in the three groups, male or female, in the mean levels of closeness attachment styles (male: *F* = 0.535, *p* = 0.658, *df* = 3; female: *F* = 2.381, *p* = 0.068, *df* = 3) and dependence attachment styles (male: *F* = 1.352, *p* = 0.256, *df* = 3; female: *F* = 3.193, *p* = 0.023, *df* = 3). However, the mean level of dependence attachment styles was lower in the Sub-type group compared to other groups, as shown in Fig. 4.

## Discussion

This study found that attachment style and role selection in BDSM were related to gender: 39.9% of the male practitioners acted as a Dom-type, and 60.5% of the female practitioners acted as a Sub-type, which is similar to the gender distribution of BDSM practitioners in other countries. In a Belgian study on BDSM, 42.9% of the male practitioners acted as a Dom-type, while 59.3% of the female practitioners acted as a Sub-type (Ten Brink et al., 2021). In a Flemish study on BDSM, 79% of Dom-type practitioners were male and 68% of Sub-type practitioners were female (Schuerwegen et al., 2021). In a study on identity selection, men were more inclined overall to choose Dom-type, while women were more inclined to choose Sub-type (Weierstall & Giebel, 2017), which seemed to be related to the partner selection preferences of the participants. Studies have shown that women are more inclined to choose partners with dominance and show a higher tendency toward sexual obedience (Giebel et al., 2015). In addition, this role tendency seems to be related to the different physiological structures of men and women and the social and cultural background of the group. Some studies have pointed out that dominant behavior is affected by sex hormones, and that a positive correlation exists between the two (Giacolini & Sabatello, 2018). Of course, there are also some women who choose Dom-type roles, which might be an attempt to find interest in a short-term relationship or to experience gender switching (Giebel et al., 2013).

This study has revealed significant differences in gender distribution among BDSM practitioners in China with regard to choosing identities, which seems to be influenced by traditional Chinese culture or even gender discrimination. In China, gender equality issues are affected by many factors, and gender stereotypes persisted for a long time before China’s collectivist culture made people more likely to voluntarily internalize gender stereotypes, while counter-stereotypes might face greater social pressure (Li et al., 2021), which potentially affects the identity selection of male and female BDSM practitioners in China (Quanbao et al., 2011).

This study also found that women reported higher levels of anxiety scores than men, which is similar to previous research by Ciocca et al. (2020), who found that women showed more anxiety attachment styles compared to men. Therefore, attachment style is also probably related to gender distribution. After controlling for gender distribution, the current study found no significant differences in the three BDSM identity groups and the control group in the mean levels of closeness types and dependence types, but found significant differences in the mean level of anxiety types in both male and female. The Sub-type had a high anxiety score, whereas the Dom-type had the lowest. The closeness score measures the individual’s comfort with proximity and intimacy; the dependence score measures the extent to which the individual is willing to depend on others when needed; and the anxiety score measures a person’s worry about being abandoned or disliked (Chi et al., 2016). Although there was no significant difference among the BDSM identity groups and the control group in the mean levels of closeness and dependence, all respondents did not reach a high level, indicating that the respondents were not likely to want proximity or to depend on others as a whole. Because of the influence of Asian culture, such as the Confucian moral system and collectivist beliefs in China, it is likely that the respondents in the current study were more inclined to pragmatic interpersonal relationships compared to the Western groups in previous studies (Zeng et al., 2016). This could explain the finding that respondents were more inclined not to establish close or dependent relationships with others, thus showing slightly low mean levels of closeness and dependence attachment styles.

Compared to Dom-type practitioners, anxiety scores were higher in the Sub-type group, Switch type group, and control group. A low anxiety score means that individuals do not have a high level of concern about partner separation. They believe that they can provide a sense of security to their partner, and also believe that their partner will actively approach and rely on them, which is the same as the requirements of the Dom-type identity in BDSM. Dom-type practitioners share similarities with the “Machiavellian” (Inancsi et al., 2015). The Machiavellian displays control over their partner, are unable to rely on their partner due to an aversion to uncertainty, and show no anxiety about the separation itself, but anger at the inability to control the partner (Inancsi et al., 2015). It has been speculated that Dom-type practitioners, as executors of power, are also insensitive to separation anxiety. Therefore, BDSM practitioners who show lower scores on attachment anxiety may be inclined to act as a Dom-type. At the same time, individuals with low levels of separation anxiety would have a higher sense of self-identity and expect to have control over their partners. Dom-type practitioners hold most of the power in BDSM activities, being able to propose separation in a BDSM relationship but also to provide a sense of security to Sub-type practitioners. Thus, in the current study, acting as a Dom-type was favored by BDSM practitioners with lower anxiety scores.

Sub-type practitioners showed the highest level of anxiety scores, which raises the question: why do individuals with high levels of anxiety about separation choose a Sub-type identity? First, it is necessary to clarify the reason why Sub-type practitioners choose to exchange power. Human behavior and even psychology are heavily influenced by power relations. Human society operates under rules of “discipline and punishment.” Under such a social background, groups begin to rely on discipline. Sub-type practitioners, being dependent on discipline, might show higher dependence on discipline than other groups, and may even feel insecure when they do not receive discipline. It can be concluded that, for Sub-type practitioners, a sense of security often comes from the restraint and control provided by their partners. In addition, Sub-type practitioners are required to transfer part of their power to their partners in BDSM activities. The initiative is wholly controlled by their partners; once separated, Sub-type practitioners cannot meet their expectations of being controlled. Therefore, separation is unacceptable to Sub-type practitioners. At the same time, Sub-type practitioners might increase their self-examination in order to gain partner approval. If they perceive that they have not met their partner’s expectations, the result might be an increase in their fear of being separated, which is consistent with the performance of individuals with high anxiety scores.

Certain limitations to this study must be noted. Firstly, the age range of the majority of respondents was 18–30 years, which is not representative of other age groups. Secondly, the gender representation was unbalanced, which affects the representativeness of the data to some extent, i.e., the results may not be applicable to all BDSM populations. Thirdly, the questionnaire was designed for a Chinese population; therefore, the results are only representative of BDSM practitioners and non-BDSM practitioners in China. Fourthly, the study was a cross-sectional study; therefore, it could not perform a direct causal analysis of the factors involved in driving BDSM identity.

The results demonstrate that attachment style is a psychological mechanism related to BDSM identity, and that gender is also related to BDSM identity, but further research related to the psychological processes that are inspirations for BDSM identity are necessary. This would help BDSM practitioners to better understand the characteristics of different BDSM identities and to choose suitable partners, which would also reduce the occurrence of negative experiences.

## Adult Attachment Scale

The following instructions were provided to the participants in this study, with their responses measured on a five-point scale (1: strongly disagree; 2: disagree; 3: neutral; 4: agree; and 5: strongly agree): “Please read the sentences below which are designed to measure how you feel about relationships. Consider all of your relationships (past and present) and answer questions about what you usually feel in these relationships. If you have never entered an affective relationship, choose the option that matches your feelings based on how you think the affection will be.”

**Table:**

I find it relatively easy to get close to my partner
I find it difficult to allow myself to depend on romantic partners
I often worry that my partner doesn’t really love me
I find that partners won’t get as close to me as I want
I feel comfortable with dependence on romantic partners
I don’t care if someone is too close to me
I find that when I need help, nobody is there for me
I am nervous when partners get too close to me
I worry that romantic partners won’t care about me as much as I care about them
When I show my feelings for romantic partners, I’m afraid they will not feel the same about me
I often wonder whether my partner really cares about me
I am very comfortable being close to romantic partners
I don’t feel comfortable opening up to romantic partners
I know that when I need help, there’s always someone there
I want to be close to others, but I worry about hurting myself
I find it hard to trust others completely
I can’t be sure that I can always find someone I can depend on when I need them
I get uncomfortable when a romantic partner wants to be very close

## References

[CR1] Alison L, Santtila P, Sandnabba NK, Nordling N (2001). Sadomasochistically oriented behavior: Diversity in practice and meaning. Archives of Sexual Behavior.

[CR2] Brennan KA, Clark CL, Shaver PR, Simpson JA, Rholes WS (1998). Self-report measurement of adult attachment: An integrative overview. Attachment theory and close relationships.

[CR3] Brown A, Barker ED, Rahman Q (2020). A systematic scoping review of the prevalence, etiological, psychological, and interpersonal factors associated with BDSM. Journal of Sex Research.

[CR4] Carlström C (2019). BDSM, becoming and the flows of desire. Culture, Health and Sexuality.

[CR5] Chi, X., Zhang, P., Wu, H., & Wang, J. (2016). Screening for postpartum depression and associated factors among women in China: A cross-sectional study. *Frontiers in Psychology*, *7*. 10.3389/fpsyg.2016.01668 10.3389/fpsyg.2016.01668 PMC5088192 27847483

[CR6] Ciocca G, Zauri S, Limoncin E, Mollaioli D, D’Antuono L, Carosa E, Nimbi FM, Simonelli C, Balercia G, Reisman Y, Jannini EA (2020). Attachment style, sexual orientation, and biological sex in their relationships with gender role. Sexual Medicine.

[CR7] Coleman E, Rahm-Knigge RL, Danielson S, Nielsen KH, Gleason N, Jennings T, Miner MH (2023). The relationship between boredom proneness, attachment styles and compulsive sexual behavior. Journal of Sex and Marital Therapy.

[CR8] Collins NL, Read SJ (1990). Adult attachment working models, and relationship qualicy in daring couples. Journal of Personality and Social Psychology.

[CR9] Darban F, Safarzai E, Koohsari E, Kordi M (2020). Does attachment style predict quality of life in youth? A cross-sectional study in Iran. Health Psychology Research.

[CR10] De Neef N, Coppens V, Huys W, Morrens M (2019). Bondage-discipline, dominance-submission and sadomasochism (BDSM) from an integrative biopsychosocial perspective: A systematic review. Sexual Medicine.

[CR11] Giacolini T, Sabatello U (2018). Psychoanalysis and affective neuroscience. The motivational/emotional system of aggression in human relations. Frontiers in Psychology.

[CR12] Giebel G, Moran J, Schawohl A, Weierstall R (2015). The thrill of loving a dominant partner: Relationships between preference for a dom-inant mate, sensation seeking, and trait anxiety. Personal Relationships.

[CR13] Giebel G, Weierstall R, Schauer M, Elbert T (2013). Female attraction to appetitive-aggressive men is modulated by women’s menstrual cycle and men’s vulnerability to traumatic stress. Evolutionary Psychology.

[CR14] Hoenicka MA, López-de-la-Nieta O, Martínez Rubio JL, Shinohara K, Neoh MJ, Dimitriou D, Esposito G, Iandolo G (2022). Parental bonding in retrospect and adult attachment style: A comparative study between Spanish, Italian and Japanese cultures. PLoS ONE.

[CR15] IInáncsi T, Láng A, Bereczkei T (2015). Machiavellianism and adult attachment in general interpersonal relationships and close relationships. Europe’s Journal of Psychology.

[CR16] Jie B, Jia L (2017). Exploring power exchange of BDSM. Chinese Journal of Human Sexuality.

[CR17] Jozifkova, E. (2013). Consensual sadomasochistic sex (BDSM): The roots, the risks, and the distinctions between BDSM and violence. *Current Psychiatry Reports,**15*. 10.1007/s11920-013-0392-1 10.1007/s11920-013-0392-1 23933978

[CR18] Li J, Liu Y, Song J (2021). The relationship between gender self-stereotyping and life satisfaction: The mediation role of relational self-esteem and personal self-esteem. Frontiers in Psychology.

[CR19] Mortazavizadeh Z, Göllner L, Forstmeier S (2022). Emotional competence, attachment, and parenting styles in children and parents. Psicologia, Reflexão e Critica: Revista Semestral Do Departamento de Psicologia Da UFRGS.

[CR20] Palmer BA, Richardson EJ, Heesacker M, DePue MK (2018). Public stigma and the label of gambling disorder: Does it make a difference?. Journal of Gambling Studies.

[CR21] Pietromonaco PR, Barrett LF (1997). Working models of attachment and daily social interactions. Journal of Personality and Social Psychology.

[CR22] Quanbao J, Shuzhuo L, Marcus WF (2011). Demographic consequences of gender discrimination in China: Simulation analysis of policy options. Population Research and Policy Review.

[CR23] Richters J, de Visser RO, Rissel CE, Grulich AE, Smith AMA (2008). Demographic and psychosocial features of participants in bondage and discipline, "Sadomasochism" or dominance and submission (BDSM): Data from a national survey. Journal of Sexual Medicine.

[CR24] Schori A, Jackowski C, Schön CA (2022). How safe is BDSM? A literature review on fatal outcome in BDSM play. International Journal of Legal Medicine.

[CR25] Schuerwegen A, Huys W, Coppens V, De Neef N, Henckens J, Goethals K, Morrens M (2021). The psychology of kink: A cross-sectional survey study investigating the roles of sensation seeking and coping style in BDSM-related interests. Archives of Sexual Behavior.

[CR26] Sheinbaum T, Kwapil TR, Ballespí S, Mitjavila M, Chun CA, Silvia PJ, Barrantes-Vidal N (2015). Attachment style predicts affect, cognitive appraisals, and social functioning in daily life. Frontiers in Psychology.

[CR27] Stein, D. (1984). *Safe sane consensual: The evolution of a shibboleth*. GMSMA Committee.

[CR28] Szielasko AL, Symons DK, Lisa Price E (2013). Development of an attachment-informed measure of sexual behavior in late adolescence. Journal of Adolescence.

[CR29] Ten Brink S, Coppens V, Huys W, Morrens M (2021). The psychology of kink: A survey study into the relationships of trauma and attachment style with BDSM interests. Sexuality Research and Social Policy.

[CR30] Tucker JS, Rodriguez A, Davis JP, D’Amico EJ (2022). Cross-lagged associations of insecure attachment style, alcohol use, and sexual behavior during emerging adulthood. Archives of Sexual Behavior.

[CR31] Weierstall R, Giebel G (2017). The sadomasochism checklist: A tool for the assessment of sadomasochistic behavior. Archives of Sexual Behavior.

[CR32] Wei-li W, Wei Z, Xie-he L (2004). The reliability and validity of Adult Attachment Scale (AAS-1996 revised edition): A report on its application in China. Journal of Sichuan University.

[CR33] Wignall L, McCormack M (2017). An exploratory study of a new kink activity: “Pup play”. Archives of Sexual Behavior.

[CR34] Wismeijer AA, Van Assen MA (2013). Psychological characteristics of BDSM practitioners. Journal of Sexual Medicine.

[CR35] Zeng XL, Pan YQ, Zhou H, Yu S, Liu XP (2016). Exploring different patterns of love attitudes among Chinese college students. PLoS ONE.

